# Analysis of COVID-19-Related User Content on the Baseball Bulletin Board in 2020 through Text Mining

**DOI:** 10.3390/bs13070551

**Published:** 2023-07-02

**Authors:** Shang-Chun Ma, Ching-Ya Su, Sheng-Fong Chen, Shintaro Sato, Shang-Ming Ma

**Affiliations:** 1 Institute of Physical Education, Health and Leisure Studies, National Cheng Kung University, No. 1, Daxue Road, East District, Tainan 701401, Taiwan; 10102009@gs.ncku.edu.tw (S.-C.M.); rb6084077@gs.ncku.edu.tw (C.-Y.S.); 2 Department of Recreational Sport and Health Promotion, National Pingtung University of Science and Technology, No. 1, Shuefu Road, Neipu, Pingtung 912301, Taiwan; 3 Faculty of Sport Sciences, Waseda University, 3-4-1 Higashifushimi STEP22 Nishitokyo, Tokyo 202-0021, Japan; satoshintaro@aoni.waseda.jp

**Keywords:** COVID-19, baseball, text mining, social identity, service quality, social media

## Abstract

The world engaged in online sport watching during COVID-19. Fortunately, in Taiwan, the pandemic was stably controlled in 2020, allowing for the continuation of the Chinese Professional Baseball League (CPBL); this attracted international attention and encouraged relevant discussions on social media in Taiwan. In the present study, through text mining, we analyzed user content (e.g., the concepts of sports service quality and social identity) on the Professional Technology Temple (PTT) baseball board—the largest online bulletin board system in Taiwan. A predictive model was constructed to assess PTT users’ COVID-19-related comments in 2020. A total of 422 articles and 21,167 comments were retrieved. PTT users interacted more frequently during the closed-door period, particularly during the beginning of the CPBL in April. Effective pandemic prevention, which garnered global attention to the league, generated a sense of national identity among the users, which was strengthened with the development of peripheral products, such as English broadcasting and live broadcasting on Twitch. We used machine learning to develop a chatbot for predicting the attributes of users’ comments; this chatbot may improve CPBL teams’ understanding of public opinion trends. Our findings may help stakeholders develop tailored programs for online spectators of sports during pandemic situations.

## 1. Introduction

During COVID-19, Taiwan garnered international attention because of its effective pandemic preventive strategies. This increases the possibility of Taiwan receiving the right to host international sports events in the future. We noted that the fans focused on creating content on social media to promote interaction and increase engagement during the regular season closed-door period. Online engagement and display of sports-related passion may improve spectator attendance and the social identity of fans [1]. Social identity refers to individuals’ self-identification and perceived sense of belonging to a certain group as well as active engagement in group activities [2]. A sense of social identity affects individuals’ cocreation behaviors [3]. Earlier studies have focused primarily on real-world interactions and have rarely explored the concept of social identity theory in the virtual world [4]. The analysis of specific trends on social media, such as the Professional Technology Temple (PTT: the largest online bulletin board in Taiwan) may improve our understanding of relevant opinions of professional and experienced users, which may offer important insights into different topics [5]. Thus, social media appears to be a suitable setting for exploring the social identities of users in the context of specific topics. Sport service quality is closely associated with the success of sports leagues [6]. The assessment of the two key categories (core and peripheral products) of sport service quality may help us identify the association between the categories and relevant social media content. Social media provides an alternative avenue for accessing online spectators’ positive and negative feedback on sport service quality. The online activities of social media users may be assessed in terms of their contributions (number of comments) and creations (number of posts).

**Figure 1:**
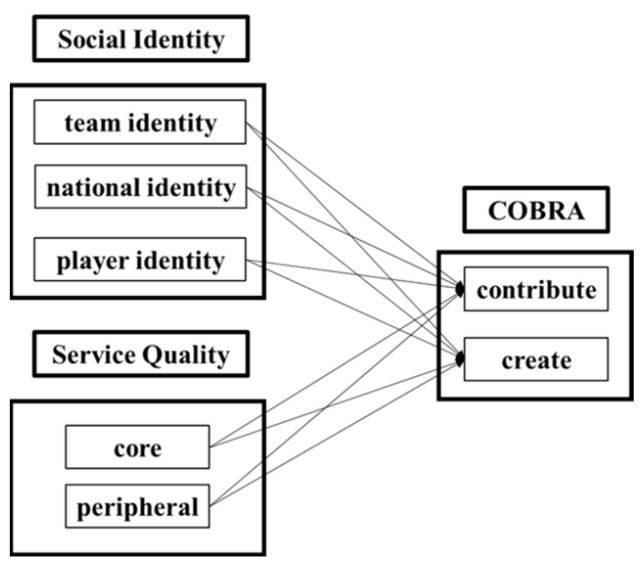
Conceptual framework.

In the present study, the social identity theory was divided into three levels: team identity, national identity, and player identity. The consumers online brand-related activities (COBRA) framework was used to explore users’ engagement behaviors on the PTT baseball board. After referring to the studies conducted by Buzeta et al. [7], Vale and Fernandes [8], Saridakis et al. [9], and Piehler et al. [10], we modified and deleted the consumption dimension of the COBRA because our aim was to evaluate the effects of users’ active behaviors on their motivation to use social media. This was an unobtrusive study; the PTT does not reveal the number of profiles or post visitors. Figure 1 depicts the conceptual framework used in our study.

Through text mining, we analyzed users’ COVID-19-related content posted on the PTT baseball board in 2020, which included the concepts of sports service quality and social identity. In addition, a predictive model was constructed to assess comment attributes on the basis of their COVID-19-related comments on PTT. Our study objectives were as follows: to investigate the influence of users’ social identity on their social media engagement, to identify the association between different categories of sport service quality and users’ social media engagement, and to use machine learning for constructing a model and a chatbot for the teams participating in the Chinese Professional Baseball League (CPBL).

## 2. Materials and Methods

### 2.1. Data Collection

Data were collected from the PTT baseball bulletin board by using PHP (hypertext preprocessor) curl and were managed using Excel. Although the baseball board covers various professional baseball leagues, such as Major League Baseball, Nippon Professional Baseball, and Korea Baseball Organization League, the number of posts on the CPBL exceeds those on others because it is the most popular league in Taiwan. In the present study, we focused particularly on CPBL-related articles to explore the cultural meaning of Taiwanese baseball. Data were extracted from relevant COVID-19-related posts made in 2020 (keywords: *epidemic*, *masks*, *new coronavirus*, *closed doors*, and *pneumonia*) on the PTT baseball board. The data set included various data, such as post times, classification, post topics, post texts, and comments (three attributes [reactions]: *boo*, *like*, and *neutral*).

### 2.2. Multivariate Regression Analysis

SPSS (version 25.0) was used to analyze the number of post days and the classification of posts using descriptive statistics. Data regarding the uses and gratifications (U&G) classification, model concepts, comment attributes, and post period were used in a multivariate regression analysis.

#### 2.2.1. Classification

Original posters classify their content on the basis of topics before posting on the PTT. We classified the articles into the information, interaction, and entertainment categories of the U&G classification according to comment attributes.

#### 2.2.2. Concepts

The articles were classified according to the keywords used in relevant studies. Sport service quality was divided into two categories: core and peripheral products. Furthermore, social identity was divided into three levels: team identity, player identity, and national identity.

#### 2.2.3. Comment Attributes

On the PTT, comments are evaluated using three reactions: boo, like, and neutral. These reactions correspond to those of *dislike*, *like*, and *no opinion*, respectively, on other social media (e.g., Facebook).

#### 2.2.4. Post Period

The CPBL in 2020 was divided into four periods: preseason (before the league), regular season closed door (closed-door games, audience not allowed), regular season open door (audience allowed), and nonseason (after the 2020 season).

### 2.3. Modeling and Evaluation

**Figure 2:**
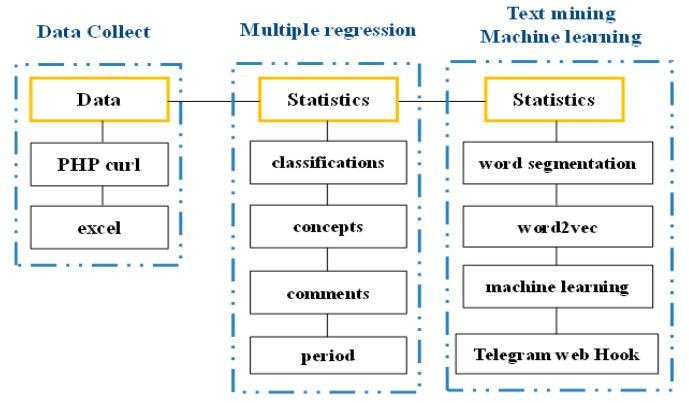
Flowcharts for data collection, text mining, and machine learning.

We used PHP for word segmentation and Word2vec for constructing a machine learning model. For different comment attributes, word segmentation was performed to convert sentences into words. Then, the words were processed using Word2vec to identify word vectors. Next, the predictive model was constructed, which could predict comment attributes. Finally, a chatbot was developed using Telegram Webhooks (Figure 2).

## 3. Results

### 3.1. Descriptive Statistics

We retrieved a total of 422 articles and 21,167 comments from the PTT baseball board. Table 1 summarizes the descriptive statistics of the contribution (number of comments) and creation (the number of posts) of PTT users in the contexts of social identity, sport service quality, and post period. Among the three attributes, *like* had the highest percentage in the contexts of social identity, sport service quality, and post period. Regarding social identity, the highest proportion of the articles was related to national identity (87%) and comments (76%). Regarding sport service quality, the highest proportion of articles was related to peripheral sport service quality (58%) and comments (55%). Regarding post period, the highest proportion of articles was related to regular season closed door (42%) and comments (54%).

We determined the monthly numbers of articles stratified by concept and collected data regarding the number of confirmed cases of COVID-19 in Taiwan in 2020 (Table 2) [11]. The top three months with the highest numbers of confirmed cases were March, November, and December. Regarding user content, discussions were focused primarily on peripheral sport service quality and national identity. The number of posts was the highest in April.

### 3.2. Results of Multivariate Regression Analysis

Post classification, social identity, sport service quality, and post period were regarded as independent variables in this study, whereas the three comment attributes were regarded as dependent variables. Chatting, team identity, peripheral sport service quality, and regular season closed door served as reference variables for post classification, social identity, sport service quality, and post period, respectively. The value of collinearity tolerance was >0.2 and the variance inflation factor was <5, indicating no collinearity-related problems.

Table 3 summarizes the statistics related to post classification. Under the concept of information, we evaluated the following variables: news, intelligence, sharing, and home run. News and intelligence (information concept) exerted nonsignificant effects on the boo (news: *β* = −0.009 and *p* > 0.05; intelligence: *β* = −0.008 and *p* > 0.05), like (news: *β* = −0.056 and *p* > 0.05; intelligence: *β* = 0.018 and *p* > 0.05), and neutral (news: *β* = 0.013 and *p* > 0.05; intelligence: *β* = 0.041 and *p* > 0.05) attributes. Sharing (information concept) exerted significantly positive, significantly positive, and nonsignificant effects on the boo (*β* = 0.135; *p* < 0.01), like (*β* = 0.142; *p* < 0.01), and neutral (*β* = 0.077; *p* < 0.01) attributes, respectively. Furthermore, home run exerted nonsignificant, significantly positive, and nonsignificant effects on the boo (*β* = −0.012; *p* > 0.05), like (β = 0.29; *p* < 0.01), and neutral (*β* = 0.05; *p* < 0.01) attributes, respectively.

Under the concept of interaction, we evaluated the following variables: questionnaires and discussions. These variables exerted nonsignificant effects on the boo (questionnaires: *β* = 0.012 and *p* > 0.05; discussions: *β* = 0.063 and *p* > 0.05), like (questionnaires: *β* = −0.038 and *p* > 0.05; discussions: *β* = −0.052 and *p* > 0.05), and neutral (questionnaires: *β* = −0.048 and *p* > 0.05; discussions: *β* = 0.06 and *p* > 0.05) attributes.

Under the concept of entertainment, we evaluated the money variable. This variable exerted nonsignificant, significant, and significant effects on the boo (*β* = −0.022; *p* > 0.05), like (*β* = 0.389; *p* < 0.01), and neutral (*β* = 0.03; *p* > 0.05) attributes, respectively.

Table 4 presents the statistics related to social identity. National identity exerted nonsignificant, significantly positive, and nonsignificant effects on the boo (*β* = 0.034; *p* > 0.05), like (*β* = 0.093; *p* < 0.01), and neutral (*β* = −0.019; *p* > 0.05) attributes, respectively. Player identity exerted nonsignificant effects on the boo (*β* = −0.024; *p* > 0.05), like (*β* = −0.043; *p* > 0.05), and neutral (*β* = −0.065; *p* > 0.05) attributes.

Table 5 presents the statistics related to sports service quality. Core sport service quality exerted nonsignificant, significant, and nonsignificant effects on the boo (*β* = −0.051; *p* > 0.05), like (*β* = −0.001; *p* < 0.05), and neutral (*β* = 0.01; *p* > 0.05) attributes, respectively.

Table 6 presents the statistics related to post period. Regarding the boo attribute, preseason exerted significant effects on the boo (*β* = −0.134; *p* < 0.05), like (*β* = −0.061; *p* < 0.05), and neutral (*β* = −0.077; *p* < 0.05) attributes. Regular season open door exerted nonsignificant, significant, and nonsignificant effects on the boo (*β* = −0.073; *p* > 0.05), like (*β* = −0.063; *p* < 0.05), and neutral (*β* = −0.108; *p* > 0.05) attributes, respectively. Furthermore, nonseason exerted nonsignificant effects on the boo (*β* = −0.046; *p* > 0.05), like (*β* = −0.008; *p* > 0.05), and neutral (*β* = −0.063; *p* > 0.05) attributes.

### 3.3. Machine Learning and Model Prediction

#### 3.3.1. Basic Training

First, the data were curled using PHP. Second, natural language processing—word segmentation and Word2vec—was performed to generate training files. Third, machine learning was used to construct a predictive model, which could predict comment attributes by finding similar texts in the training files. Finally, the chatbot was developed using Telegram Webhooks.

#### 3.3.2. System Implementation

When users input texts or sentences into the chatbot system on Telegram, the chatbot can directly respond to the comment attributes. For example, if a user inputs “*precaution*” into the chatbot, the system will generate an output of “like”. Furthermore, if one puts “*If you have enough inferiority complex, don’t go out anymore. You are embarrassed anyway*” into the chatbot, the system will generate an output of “boo”.

If the inputted text has different attributes but the same text ranking, the system will automatically use the most frequent attribute as the main attribute. The appendix presents the text contents of different comment attributes to explain the output generation by the chatbot.

## 4. Discussion

### 4.1. Theoretical Implications

The Internet promotes social gratification [12]. In the era of consumer-led marketing, the COBRA can help official stakeholders understand consumers social media [13]. The manner in which consumers use social media is highly interactive and connected [14] and may help develop public opinions [15]. Text mining can help us understand users’ real opinions [16,17].

We used social identity and sport service quality to understand COBRA engagement; this venture is new in the domain of sports research. The chatbot developed in the present study may help official stakeholders better understand the attributes of users’ comments on the PTT baseball board during COVID-19.

We found that articles with a higher number of posts attracted more comments. Therefore, contribution may be positively correlated with creation. Consumers’ interactions on social media increased gradually, shifting from consumption to contribution. Contribution is the key to future post creation [14]. Interaction positively affects the relationship between official stakeholders and consumers, and consumer engagement affects loyalty [18].

#### 4.1.1. Social Identity

National identity was associated with the highest levels of contribution and creation. The content of national identity involved the facts that 2020 CPBL was the first baseball league organized after the COVID-19 outbreak and was broadcast globally; these factors were ascribed to Taiwan’s effective and successful pandemic prevention measures. This invoked a sense of national pride and the *like* comments. The words *global*, *world*, *English*, *rename*, and *leave a name* attracted a high number of like comments, indicating that users wanted to advertise the CPBL to the world as an expression of their national identity. These findings are different from those of earlier studies [19,20] ascribing national identity to a satisfactory performance of the national team, the participation of Taiwanese players in professional leagues abroad, and the efficient hosting of international sporting events. In the present study, the in-depth exploration of online content revealed that sport fans’ (users’) national identity was attributed to the country’s effective pandemic prevention. Thus, the CPBL became an outlet for sports fans to demonstrate their national identity. The CPBL must be made aware of the fact that offline fans are likely to share external social identities (e.g., player and team identities) with online fans, which helps expand the fan base [21]. Thus, the CPBL must promote national identity to effectively encourage its fans to attend the games in the coming season [22].

#### 4.1.2. Sport Service Quality

Peripheral sport service quality was associated with the highest levels of contribution and creation. This category of sport service quality more easily generated *like* comments than the other category, core sport service quality. Words such as *baseball*, *English*, *chat room*, *cheerleading*, *world*, *global*, *fans*, and *epidemic prevention* were highly frequent in the context of peripheral sport service quality. The word baseball was found in repost articles for politicians and fans anticipating live games. The word chatroom received no boo comments and approximately 80% of all like comments. The remaining words were mostly associated with positive comments. Therefore, in 2020, PTT users had positive attitudes toward peripheral sport service quality; this finding is consistent with that reported by Tan and Lee [23] who indicated the reputation of Asian baseball improved during COVID-19 because it was the only live sports event during the pandemic.

The results of the frequency analysis revealed that *baseball*, *world*, *global*, and *rename* had high frequency in the context of core sport service quality. The word *baseball* received the highest numbers of both like and boo comments. Specifically, the like comments were associated with the game schedule and players, which indicated users’ support for the schedule and player performances. The words *global* and *world* also received like comments, which indicated the users’ intention to promote the CPBL; by contrast, *rename* received boo comments. *Rename* referred to adding Taiwan to the name CPBL. Thus, discussions on core sport service quality may help promote the CPBL. Furthermore, combining discussions on core sport service quality with those on national identity may help reduce the number of boo comments. The rationale of the aforementioned strategy is similar to that mentioned by Chiang and Chen [24], who reported that integrating national identity and national morale through sports recognized by other nations may help develop positive attitudes in Taiwanese individuals. Core sport service quality is a strong driver of consumer behaviors [25]; the unpredictability of sports enhances consumer focus on the core of the competition [26]. Our findings indicate the importance of core sport service quality, which attracted the highest numbers of both like and boo comments. It is a double-edged sword. Our study may offer important insights to the CPBL.

Overall, focusing on both core and peripheral sport service qualities online is essential because this represents a promising avenue for obtaining international consumers [27]. The influence of online fans is extensive and global; hence, professional sport leagues must understand online fans’ positive and negative feedback and respond immediately. The leagues’ brand names can be promoted through core and peripheral products to improve the fans’ consumption behaviors [25].

#### 4.1.3. Post Classification

The articles related to *home run*, *sharing*, and money were more likely to attract like comments than those related to chat. This indicates that the affinity of users toward articles on players’ home run records, information shared from other platforms, and original posters encouraging users is more important than merely expressing personal opinions. These findings are similar to those reported by Muntinga et al. [13] who found that social media users are driven by entertainment, which is positively correlated with user satisfaction.

Articles related to *sharing* were more likely to receive boo comments than those related to *chat*. As information articles describe events from an objective perspective, both positive and negative public opinions are expected. Sharing is essential for establishing a relationship between official stakeholders and fans [28]. However, analysis of the content of articles related to sharing that received boo comments revealed that these articles were shared by politicians and were related to baseball-focused pandemic prevention policies. Politics-related keywords that received boo comments included *cockroaches*, *politicians*, *government*, and *politics*. The negative comments indicated users’ dissatisfaction with irrelevant information on the baseball board. Sharing helps promote users’ social experience and encourage them to browse and comment on posts [29]. Team managers should avoid or restrict irrelevant posts on their social media to reduce the number of negative comments. The topics of articles related to chat that received boo comments included pandemic prevention policies for the regular season open-door period. Although users expressed their opinions through chats, they were actively and seriously involved in relevant discussions and thus received fewer negative comments. Interaction is regarded the core of audience activities, which can improve our understanding of the requirements of social media and their users [30]. The marketing managers of sports teams must create interaction boards on team social media to explore the requirements of their fans, as encouraging discussions on crucial issues may promote the active engagement of users and reduce the number of negative comments.

#### 4.1.4. Chatbot’s Significance

The chatbot developed in our study predicts comment attributes. Earlier studies on the use of big data in sports management focused on word frequency [31,32,33,34] and topic identification [5,28,35]. By contrast, we developed the predictive model using machine learning; this innovative model may help advance sport research. Using this chatbot, professional baseball teams can effectively manage their online fanbase by better understanding user attitudes and reacting immediately and appropriately.

### 4.2. Practical Implications

Using both qualitative and quantitative methods in the present study, we assessed online COVID-19-related sports content through text mining. Through the thematic analysis of words with high frequency, key points can easily be identified from an unstructured content. We found that the highest number of posts and comments were made in the regular season closed-door period. This implies that most sports fans were eager for elevated levels of social interactions through virtual platforms such as social media (e.g., PTT). Therefore, sports fans may not abandon their favorite teams even if they are temporarily not allowed to enter the stadium to watch live matches. This is a key implication for the marketing managers of sports teams. They should invest in intensive marketing and building or managing a relationship with their fans through social media [36].

Core products are a predominant and indispensable part of competitions. However, during COVID-19, a relatively high proportion of posts were related to peripheral products (i.e., English broadcasts and Twitch). The availability of English broadcasts and various platforms for the CPBL may help promote users’ national identity. In the present study, the national identity of PTT users was identified to be a strong driver of like comments, which, in turn, promotes sports leagues through word of mouth. The chatbot developed in our study can help understand the posts and comments during the pandemic period. It can also help the CPBL teams to predict public opinions and act accordingly.

### 4.3. Limitations and Future Directions

Our study has some limitations. First, we focused only on COVID-19-related content posted in 2020. Thus, this study does not cover widespread issues that may attract readers’ attention to professional baseball and COVID-19. For a comprehensive analysis of user content on the PTT baseball board, future studies should collect data chronologically to constitute both training and validation data sets. Thus, the model fit and accuracy of our model may be improved in future studies.

Second, the collection of data focusing on incidents [37] and new theories (i.e., cocreation) may help us better understand users’ interactions [38] and emotions to improve the representativeness and creativity of our model. This requires further studies.

Third, the PTT is a platform purely for text communication. Researchers are encouraged to collect data from social media, such as Facebook and Instagram, which contain pictures and videos for constructing predictive models that may enhance interaction and entertainment [39].

Fourth, understanding emotions underlying Chinese texts through text mining is challenging. The systematic calculation of emotion scores and the improvement of word segmentation accuracy may enhance the substantiveness of future studies [40].

Finally, people can freely create content on the Internet; thus, fake articles and comments cannot be entirely excluded [41]. Texts with extremely high frequency may be deleted to enhance the credibility of future studies.

### 4.4. Conclusions

With time and technological advances, the development of Web2.0 has ushered us into a whole new world—the virtual world, a platform for networking. Due to the interactivity and immediacy offered by such platforms, users become socially connected and creative consumers. Virtual networks enable social media users to contribute to the development of online public opinions through social interactions; thus, such networks may facilitate targeted problem solving and benefit organizations. The PTT has a strong influence in Taiwan. The original posters of the PTT are mostly professional and experienced fans; this factor strengthens our findings. The concept of PTT comment attributes is the same as that of upvote and downvote on Reddit; hence, the comment attributes indicate users’ attitudes toward articles posted on PTT.

Text mining may help reflect users’ real opinions because it can accurately grasp information by organizing a substantial amount of data. The three comment attributes and concepts (i.e., social identity and sport service quality) were assessed using a word cloud; this would improve professional sport leagues’ understanding of online user content and help them effectively manage their online fanbase. Furthermore, predictive models constructed using big data may help predict user opinions, which may facilitate the management of the relationship between sports teams and fans. Team managers can strengthen their fan base by posting articles to attract positive comments and rapidly resolving and clarifying negative comments.

## Figures and Tables

**Table 1:** Descriptive statistics of the COBRA model.

Comment Evaluation		Contributing				Creating
		Boo	Like	Neutral	All	-
Social identity	National	1165 (12%)	5511 (59%)	2711 (29%)	9387 (76%)	141 (87%)
	Player	88 (5%)	1406 (73%)	426 (22%)	1920 (15%)	11 (7%)
	Team	60 (5%)	726 (66%)	322 (29%)	1108 (9%)	10 (6%)
Total		-	-	-	12,415 (100%)	162 (100%)
Service quality	Core	922 (11%)	4751 (54%)	3101 (35%)	8774 (45%)	171 (42%)
	Peripheral	1417 (13%)	5424 (50%)	4029 (37%)	10,870 (55%)	239 (58%)
Total		-	-	-	19,644 (100%)	410 (100%)
Period	preseason	581 (9%)	3412 (50%)	2804 (41%)	6797 (32%)	163 (39%)
	close door	1550 (14%)	6225 (55%)	3559 (31%)	11,334 (54%)	177 (42%)
	Open	329 (14%)	1193 (55%)	893 (31%)	2415 (11%)	66 (16%)
	nonseason	54 (9%)	373 (60%)	194 (31%)	621 (3%)	16 (4%)
Total		-	-	-	21,167 (100%)	422 (100%)

Each concept in each row totals 100%.

**Table 2:** Descriptive statistics of the monthly numbers of articles (stratified by core concepts) and confirmed COVID-19 cases in 2020.

Concept	Sport Service Quality		Social Identity			Number of Confirmed Cases
Month	Core	Peripheral	National	Player	Team	
Jan	2 (1%)	1	0	0	0	19 (2%)
Feb	5 (3%)	9 (5%)	0	0	0	26 (3%)
Mar	33 (19%)	62 (25%)	1 (1%)	3 (11%)	0	330 (41%)
Apr	106 (61%)	89 (36%)	120 (84%)	22 (78%)	19 (85%)	61 (8%)
May	6 (4%)	56 (23%)	14 (10%)	2 (7%)	0	9 (1%)
Jun	1 (1%)	4 (2%)	0	0	0	6 (1%)
Jul	3 (2%)	2 (1%)	0	0	1 (5%)	29 (4%)
Aug	2 (1%)	10 (4%)	1 (1%)	0	1 (5%)	16 (2%)
Sep	2 (1%)	1	1 (1%)	0	0	25 (3%)
Oct	2 (1%)	6 (2%)	1 (1%)	1 (4%)	0	52 (7%)
Nov	6 (4%)	5 (2%)	3 (2%)	0	0	116 (15%)
Dec	3 (2%)	0	0	0	1 (5%)	104 (13%)
Total	171	245	141	28	22	793

**Table 3:** Regression statistics related to post classification.

Attribute	U&G Concept	Type	*β*	*p*	Tolerance	VIF
Boo	Information	News	−0.009	0.85	0.77	1.30
		Intelligence	−0.008	0.82	0.97	1.03
		Home run	−0.012	0.80	0.96	1.04
		Sharing	0.135 **	0.01	0.85	1.17
	Interaction	Questionnaires	0.012	0.94	0.92	1.09
		Discussions	0.063	0.30	0.82	1.23
	Entertainment	Money	−0.022	0.72	0.99	1.01
Like	Information	News	−0.056	0.24	0.77	1.30
		Intelligence	0.018	0.78	0.97	1.03
		Home run	0.290 ***	0.00	0.96	1.04
		Sharing	0.142 ***	0.00	0.85	1.17
	Interaction	Questionnaires	−0.038	0.22	0.92	1.09
		Discussions	−0.052	0.16	0.82	1.23
	Entertainment	Money	0.389 ***	0.00	0.99	1.01
Neutral	Information	News	0.013	0.76	0.30	0.76
		Intelligence	0.041	0.40	0.85	0.40
		Home run	0.050	0.35	0.94	0.35
		Sharing	0.077	0.12	1.55	0.12
	Interaction	Questionnaires	−0.048	0.32	−1.00	0.32
		Discussions	0.060	0.29	1.07	0.29
	Entertainment	Money	0.030	0.56	0.58	0.56

** *p* < 0.01; *** *p* < 0.001. Reference: chatting. VIF, variance inflation factor.

**Table 4:** Regression statistics related to social identity.

Attribute	Social Identity	*β*	*p*	Tolerance	VIF
Boo	National identity	0.034	0.08	0.99	1.01
	Player identity	−0.024	0.78	0.99	1.01
Like	National identity	0.093 ***	0.00	0.99	1.01
	Player identity	−0.043	0.62	0.99	1.01
Neutral	National identity	−0.019	0.37	0.99	1.01
	Player identity	−0.065	0.49	0.99	1.01

*** *p* < 0.001. Reference: team identity.

**Table 5:** Regression statistics related to sport service quality.

Attribute	Service quality	*β*	*p*	Tolerance	VIF
Boo	Core	−0.051	0.90	1.00	1.00
Like	Core	−0.001 *	0.02	1.00	1.00
Neutral	Core	0.010	0.29	1.00	1.00

* *p* < 0.05. Reference: peripheral sport service quality.

**Table 6:** Regression statistics related to post period.

Attribute	Period	*Β*	*p*	Tolerance	VIF
Boo	Preseason	−0.134 **	0.01	0.85	1.18
	Regular season (open)	−0.073	0.16	0.86	1.16
	Nonseason	−0.046	0.26	0.95	1.05
Like	Preseason	−0.061 ***	0.00	0.85	1.18
	Regular season (open)	−0.063 **	0.01	0.86	1.16
	Nonseason	−0.008	0.30	0.95	1.05
Neutral	Preseason	−0.077 *	0.05	0.85	1.18
	Regular season (open)	−0.108	0.19	0.86	1.16
	Nonseason	−0.063	0.52	0.95	1.05

* *p* < 0.05; ** *p* < 0.01; *** *p* < 0.001. Reference: regular season closed-door period.

## Data Availability

The data presented in this study are available on reasonable request from the corresponding author.
